# Impact of Nintendo Wii Games on Physical Literacy in Children: Motor Skills, Physical Fitness, Activity Behaviors, and Knowledge

**DOI:** 10.3390/sports4010003

**Published:** 2016-01-15

**Authors:** Amanda M. George, Linda E. Rohr, Jeannette Byrne

**Affiliations:** School of Human Kinetics and Recreation, Memorial University, St. John’s, NL A1C 5S7, Canada; amg205@mun.ca (A.M.G.); jmbyrne@mun.ca (J.B.)

**Keywords:** physical literacy, children, active video games, motivation

## Abstract

Physical literacy is the degree of fitness, behaviors, knowledge, and fundamental movement skills (agility, balance, and coordination) a child has to confidently participate in physical activity. Active video games (AVG), like the Nintendo Wii, have emerged as alternatives to traditional physical activity by providing a non-threatening environment to develop physical literacy. This study examined the impact of AVGs on children’s (age 6–12, *N =* 15) physical literacy. For six weeks children played one of four pre-selected AVGs (minimum 20 min, twice per week). Pre and post measures of motivation, enjoyment, and physical literacy were completed. Results indicated a near significant improvement in aiming and catching (*p* = 0.06). Manual dexterity significantly improved in males (*p* = 0.001), and females felt significantly less pressured to engage in PA (*p* = 0.008). Overall, there appears to be some positive impact of an AVG intervention on components of physical literacy.

## 1. Introduction

Physical literacy is an important factor in child development. Developing basic skills to move around confidently at an early age is critical, enabling lifelong participation in various physical activities, including rhythmic (dance) and sports [1,2]. Physical literacy includes four main domains; physical activity behaviors, physical fitness, awareness/knowledge and understanding, and motor skills [2]. Physical activity behaviors include directly measurable daily activities, such as wearing a pedometer to track daily steps. Physical fitness encompasses factors such as cardio-respiratory fitness, flexibility, and muscular strength [2]. Awareness, knowledge, and understanding summarize how an individual thinks about physical activity and its importance [2]. The motor skills domain refers directly to an individual’s physical competency, and includes mastery of fundamental movement skills, including balance, coordination and agility. It is critical that these skills are developed for future success in physical activities [1,2].

Active video games (AVG) are popular with the general population. These games—sometimes called exergames—can simulate real life activities, sports, and dances [3]. Research exploring the impact of AVG use by children shows positive improvements in physical activity levels [4], and a reduction in sedentary behaviors [5] during leisure time. Dally (2009) has extended the benefits of AVGs for children with motor control difficulties, low self-confidence, or previous negative physical activity experiences [6]. Despite the ability of AVGs to allow individuals to engage in physical activity in the relative safety of their own homes, little is known regarding their effects on the development of physical literacy. As a potential alternative to regular physical activity, understanding the effect of AVG systems in the acquisition of physical literacy skills is important.

Numerous AVG systems are available for public purchase, each with its own set of games, unique features, benefits, and limitation. The popular Nintendo Wii uses handheld controllers fitted with motion sensors to detect players’ movements. The Nintendo Wii has a high enjoyment rating compared to other systems such as the Gamercize Power Stepper, sedentary games, and treadmill walking [7]; however, a drawback is the reduced energy expenditure during gameplay as compared to Dance Dance Revolution (DDR), Monster 4 × 4, various Gamercize and Play Station games, and the Xbox Kinect [3,8]. The use of the Nintendo Wii in research is supported by factors such as a variety of available games, low-to-moderate cost, and popularity with children. The popularity of various gaming systems fluctuates over time and is impacted by the release of new systems [9]. From 2008 to 2014, the Nintendo Wii sold 82.18 million units worldwide, compared to 68.04 million for the Xbox, and 75.27 for the PS3 [9].

Previous research indicates that girls tend to play online games for intrinsic reasons, whereas boys are more motivated by external rewards [10]. The research regarding the motivation of children to play AVGs is still new [11], but indicates that there may be several factors that influence how children play. The motivation of children is greater when playing with others, as opposed to alone [11], ultimately increasing intrinsic motivation [12]. Additionally, boys traditionally reduce extraneous movements to increase the efficiency of their game play [13]. The difference in motivation for playing AVGs may also impact boys’ style of gameplay; minimalist movements are typically more efficient and may produce higher scores, whereas larger movements more closely resemble real-world play but may not be as efficient. Overall, boys may therefore not achieve similar physical activity levels as girls due to their focus on efficiency, and ultimately high scores in the AVG.

The present study investigated the effect of AVGs (Nintendo Wii) on the physical literacy, enjoyment, motivation, and competency in children. Based on earlier research [3,4,6,14,15] it is expected that children who play AVGs will experience benefits in each of the four areas of physical literacy; physical activity behaviours (measured with pedometers), physical fitness (measured with a walk test), knowledge and awareness (measured by questionnaires) and motor skills (measured with a movement assessment). Additionally, AVG play is expected to increase children’s physical literacy, enjoyment and motivation for physical activity. Differences based on gender are expected, where boys will show greater improvements in manual dexterity [13], and girls will show greater improvements in enjoyment of physical activity [10].

## 2. Methods

### 2.1. Participants

Participants were recruited from the After School Activity Center at the Memorial University Campus Childcare Center. All boys and girls aged 6–12 who attended the Activity Center two or more days per week were invited to participate. Of the 28 children in the Center, 17 agreed to participate. In total 14 children ranged in age from 6–8 and three were between 11 and 12 years. Two of the children in the 6–8 group were excluded from the analysis (one due to not completing the intervention while the other was not available for the post-test). Ethics for this project was approved by the Interdisciplinary Committee on Ethics in Human Research, 20131719-HK. 

### 2.2. Apparatus

The physical activity intervention utilized several active video games from the Nintendo Wii system; Wii Sport, Wii Sport Resort, Wii Play and Just Dance 2. Games were chosen based on how much physical activity they promote and their ability to produce increases in heart rate [4]. Children played on one of two identical Wii systems, each attached to 40 in. TV screens. Both Wii Motion Plus controllers and the associated nunchucks were available. 

To directly measure exercise intensity during each AVG play sessions, participants wore a wrist-worn Garmin Vivofit Activity Trackers and associated chest-strap heart rate sensor. The Vivofit device recorded the heart rate and the data was subsequently uploaded to the online Garmin Connect Website. In addition, participants completed the OMNI Scales of Perceived Exertion [16] after each activity session. This data provided indirect feedback on the perceived intensity of the movement experience for the participants.

In attempt to better understand the overall physical activity behaviours, participants wore a Yamax Digi-Walker Electronic Pedometer. Each child was asked to attach the pedometer to the waistband of their pants upon waking, and wore the pedometer until bed, except during water activities (such as swimming or showering). Each participant was provided with a recording chart and was asked to complete this chart each night. Steps were recorded daily for six weeks.

To assess the knowledge and awareness component of physical literacy a variety of questionnaires were used. These questionnaires included were the Physical Activity Enjoyment Scale (PACES) [17], the Intrinsic Motivation Inventory (IMI) [18], and the Rosenberg Self-Esteem Scale (RSE) [19]. The PACES is a 16-question assessment of enjoyment levels of physical activity, with responses ranging from 1 (disagree a lot) to 5 (agree a lot). The IMI consists of 21 questions divided into four subscales; perceived competence, pressure/tension, perceived choice, and enjoyment. The RSE contains ten 5-point Likert-scale questions to assess children’s self-esteem. Overall these questionnaires provided feedback on enjoyment, perceived competency, and movement knowledge.

The Movement Assessment Battery for Children (ABC) [20] assessed manual dexterity, aiming and catching, and balance; all scales are components of the motor skill domain for physical literacy. Fitness was evaluated using the six-minute walk test (6 MWT), where the distance (meters) walked in six minutes was recorded.

### 2.3. Procedures

A pre/post-test design was followed; all domains of physical literacy were measured prior to and at the end of a 6-week intervention period. Younger children (ages 6 to 8) had the PACES, IMI and RSE questionnaires explained and read to them, and then dictated their responses; older children (ages 11 to 12) were given the option of having the questionnaires read to them or completing them independently. The two physical assessments (Movement ABC and the 6MWT) were completed in the gymnasium with the researcher demonstrating what actions were required. For the Movement ABC participants were given a practice trial before the actual assessment, and results were scored as per the official guidelines [20].

The intervention consisted of, at minimum, two weekly Nintendo Wii game sessions for 20 min or more. Children played each game in pairs. Four games were provided at each of two Wii stations—Wii Sports Resort, Wii Sports, Wii Play, and Just Dance 2—and participants self-selected in order to increase ecological validity. Participants were encouraged to do their best and have fun. Games could be changed part way through play to ensure that each participant had an opportunity to select a game. During each session, participants’ heart rates were recorded with Vivofit activity trackers and heart sensors to determine level of exertion. At the end of each session, participants completed an OMNI scale of Perceived Exertion [16] to assess how tired they felt. The length of play and games played was recorded for each play session over the 6-week period. As noted earlier pedometers were worn daily for the duration of the study.

Upon completion of the intervention, the pre-tests were repeated; each scale was administered by the same research to ensure consistency. The older children that chose to complete questionnaires on their own during the pre-test also did so during the post-test. Younger children again had the questionnaires read to them and dictated their responses.

### 2.4. Data Analysis

Heart rate data was automatically calculated on the online Garmin Connect website. The peak and average heart rate values were then extracted and analyzed in SPSS. During each session the duration and type of games played were manually recorded. Total time play for each week, as well as overall time was calculated along with number of times each game was selected by each participant.

A paired samples t-test compared change over time for the 6 MWT to assess physical fitness. Comparisons were made for all participants as well as separately for boys and girls. A regression analysis used total playtime as a predictor for changes in physical fitness. Participant’s change in exertion were examined though linear trends in heart rate data and OMNI scores. A repeated measures ANOVA analyzed pedometer data changes over time. Changes in steps taken per week were also analyzed using linear trends for each participant.

Paired samples *t*-tests evaluated questionnaire data. Correlations to test the reliability between each of the scales (IMI, RSE and PACES) at pre-test and post-test were completed. A regression, using total amount of game play as a predictor, determined the influence of amount of game play. The Movement ABC, where scales include manual dexterity, aiming and catching, and balance assessed motor skills. Paired samples t-tests looked at changes over time for all participants, as well as separately for boys and girls. Total game playtime was used to predict changes in motor skills using a linear regression analysis.

## 3. Results

### 3.1. Participants

The final sample contained 15 children (7 boys, 8 girls) aged 6 to 12 (*M* = 7.9, *SD* = 2.12). Fitness was assessed at baseline with the 6MWT. On average, the sample was below average [21], but was not significantly different than the normal range for boys, *t*(6) = −4.857, *p* = 0.869, Cohen’s *d* = 0.06, or for girls, *t*(7) = −10.825, *p* = 0.662, Cohen’s *d* = 0.15. For mean 6MWT values see Table 1. Due to differences in attendance at the Childcare Center, amount of play varied between participants. Children played an average of 13.54 times (*SD* = 2.23), for an average of 25.71 min (*SD* = 4.06) per session (see Table 2).

**Table 1:** Six minute walk test (meters) means and standard deviations.

Participants	Pre-Test	Post-Test
All (*n =* 15)	549.93 (68.82)	542.30 (75.53)
Boys (*n =* 7)	559.14 (74.94)	562.29 (90.05)
Girls (*n =* 8)	541.88 (67.08)	524.81 (60.91)

**Table 2:** Frequency and average play duration.

Variable	Total	Boys	Girls
# Times Played	13.54 (2.23)	13.57 (1.62)	13.25 (2.25)
Wii Sports Resort	12.07 (2.52)	13.28 (2.06)	11.00 (2.51)
Wii Sports	0.33 (0.49)	0.28 (0.49)	0.38 (0.52)
Wii Play	1.27 (1.94)	0.86 (0.90)	1.62 (2.56)
Just Dance 2	2.73 (2.71)	0.86 (1.07)	4.38 (2.67)
Average Minutes	25.71 (4.06)	28.07 (3.53)	23.65 (3.45)
Wii Sports Resort	27.32 (3.99)	29.36 (4.10)	25.34 (2.72)
Wii Sports	20.8 (13.16)	10.00 (7.07)	28.00 (11.27)
Wii Play	14.1(1.47)	15.31 (1.55)	13.95 (1.16)
Just Dance 2	21.98 (9.30)	25.46 (14.92)	19.45 (5.17)

Notes: Frequency of each game played, mean and standard deviation. Average play duration (min), mean and standard deviation.

### 3.2. Physical Fitness

Physical fitness was assessed with the 6MWT, and exertion was measured both directly (heart rate) and indirectly (10 point OMNI scale of perceived exertion). There was no significant differences in the distance walked by participants from pre-test to post-test, indicating that children’s fitness did not change over the study duration. 

A 2 × 6 (gender by week) repeated measures ANOVA was conducted for both average and peak heart rate; neither test revealed a significant difference in heart rate over time for boys or girls. The average (*M* = 113.50 bpm; *SD* = 10.34 bpm) and peak (*M* = 150.12 bpm; *SD* = 16.72 bpm) heart rates at the beginning of the intervention were not significantly different than the average (*M* = 114.92 bpm; *SD* = 9.41 bpm) and peak (*M* = 156.49 bpm; *SD* = 14.71 bpm) heart rates at the end of the intervention. A paired samples t-test comparing exertion from the first and last weeks of the intervention found a decrease on the OMNI scale (*M*_difference_ = 1.35, *SD*_difference_ = 1.67), *t*(13) = 3.029, *p* = 0.010, Cohen’s *d* = 0.81. A post-hoc power analysis indicated an 83% chance of detecting a significant effect at the 5% level (two-tailed). Linear trends were computed for each participant to examine the changes in heart rate and perceived exertion. There was no significant change in heart rate (average or peak) over time, but there was a significant decrease in OMNI scores, *t*(14) = −3.961, *p* = 0.001, Cohen’s *d* = 1.02. A post-hoc power analysis indicated a 96% chance of detecting a significant effect at the 5% level (two-tailed). Overall, these data suggest that although no changes were noted for actual exertion, physical fitness or heart rate, participant’s level of exertion decreased. 

### 3.3. Physical Activity Behaviors

To assess daily physical activity behaviors, participants were asked to wear pedometers for the duration of the study. Nine children (5 girls, 4 boys) complied with this requirement for at least five weeks. Data is presented in Table 3. A 2 × 5 (gender by week) repeated measures ANOVA revealed no significant differences over time for either gender. An independent samples t-test compared average steps taken by boys and girls, and found a trend towards boys being more active than girls, *t*(7) = −2.313, *p* = 0.054, Cohen’s *d* = 1.54. A post-hoc power analysis indicated a 46% chance of detecting a significant effect at the 5% level (two-tailed). Linear trends for each participant showed no significant overall changes in average daily steps.

**Table 3:** Average daily steps.

Week	All Participants	Boys (*n =* 4)	Girls (*n =* 5)
Total	10,389 (2,157)	11,887 (2526)	9192 (700)
Week 1	11,603 (3,151)	13,310 (3124)	9327 (1242)
Week 2	10,763 (2,017)	10,852 (2547)	10,645 (1560)
Week 3	10,222 (2,646)	11,068 (3391)	9094 (646)
Week 4	11,655 (2,696)	13,333 (2229)	9417 (1098)
Week 5	10,406 (2,766)	10,869 (3445)	9787 (2037)

### 3.4. Knowledge and Awareness

Motivation was assessed using the four scales of the Intrinsic Motivation Inventory (Table 4). The pressure/tension scale of the IMI indicated a positive trend for all children, suggesting that they felt less pressured after the intervention. The pressure/tension subscale of the IMI is theorized to be a negative predictor of intrinsic motivation [18]. A significant improvement was evident for girls (*t*(7) = 3.65, *p* = 0.008, Cohen’s *d* = 1.29), but not for boys. A post-hoc power analysis indicated an 88% chance of detecting a significant effect at the 5% level (two-tailed). The other scales of the IMI (choice, competence, and enjoyment) were not impacted by the intervention. There were no significant changes in self-esteem (assessed with the RSE) or in enjoyment (assessed with the PACES) over the course of the intervention (see Table 4).

**Table 4:** Pre-test and post-test enjoyment, motivation, and self-esteem scores.

Test/Group		All (*n =* 15)		Boys (*n =* 7)		Girls (*n =* 8)	
		Pre	Post	Pre	Post	Pre	Post
PACES		71.20 (5.57)	68.20 (7.21)	70.86 (6.67)	66.86 (7.60)	71.50 (4.87)	69.38 (7.15)
Rosenberg Self-Esteem		22.47 (3.54)	22.60 (3.09)	23.29 (4.07)	22.71 (3.64)	21.75 (3.10)	22.50 (2.78)
IMI	Enjoyment	44.40 (4.98)	43.07 (5.35)	42.86 (5.15)	42.00 (7.21)	45.75 (4.74)	44.00 (3.25)
	Choice	29.47 (4.41)	30.53 (4.72)	32.14 (2.34)	31.29 (3.90)	27.13 (4.55)	29.88 (5.52)
	Competence	28.80 (4.77)	28.40 (4.31)	28.00 (5.89)	27.14 (5.82)	29.50 (3.82)	29.50 (2.27)
	Pressure	14.33 (4.48)	12.73 (5.28)	13.43 (3.50)	13.86 (5.21)	15.13 (5.30)	11.75 (5.50) **

Notes: Maximum scores for each scale are as follows: Physical Activity Enjoyment Scale (PACES) = 80; Rosenberg Self-Esteem Scale (RSE) = 30; Enjoyment = 49; Choice = 35; Competence = 35; Pressure = 35; ** = *p* < 0.01. Intrinsic Motivation Inventory = IMI).

To assess the degree to which the scales are related, Pearson’s correlations were conducted (Table 5). All assessments showed a high correlation between their pre and post-test scores. This is important as it demonstrates the relationship between the three scales; although the scales measure separate concepts they are related to each other. Additionally, many of the scales were correlated with each other at both pre and post-test.

**Table 5:** Test correlations.

Time Point	Variable	Post-test					
		IMI Enjoyment	IMI Competence	IMI Choice	IMI Pressure	RSE	PACES
Pre-test	IMI Enjoyment	0.583 *	0.674 **	−0.358	0.223	0.563 *	0.514 *
	IMI Competence	0.758 **	0.651 **	0.182	0.134	0.512	0.535 *
	IMI Choice	0.014	0.253	0.592 *	0.129	−0.126	−0.127
	IMI Pressure	−0.099	0.023	−0.410	0.767 **	−0.016	−0.034
	RSE	0.474	0.737 **	0.571 *	−0.136	0.684 **	0.639 *
	PACES	0.553*	0.798 **	0.319	−0.114	0.610 *	0.648 **

Notes: Left half of the diagonal is correlations between assessments at pre-test, right half of the diagonal is correlation between assessments at post-test. Highlighted boxes along the diagonal represent the correlation between the test at pre-test and post-test.* *p* < 0.05; ** *p* < 0.01.

Regression analyses used total game play as a predictor. For the choice subscale of the IMI, there was a trend towards a significant prediction for all participants (R^2^ = 0.223, *p* = 0.076), and a significant improvement for girls (R^2^ = 0.727, *p* = 0.007). Amount of game play was not a significant predictor for any other the other scales.

### 3.5. Motor Skills

Data from the manual dexterity, aiming and catching, and balance scales of the Movement ABC were compared using a paired samples *t-*test (Table 6). Aiming and catching approached significance (*t*(14) = −2.044, *p* = 0.06, Cohen’s *d* = 1.09) for all participants. A post-hoc power analysis indicated a 48% chance of detecting a significant effect at the 5% level (two-tailed). Manual dexterity improved for boys (*t*(6) = −6.00, *p* = 0.001, Cohen’s *d* = 2.26), with younger boys (*p* = 0.016, Cohen’s *d* = 2.45) showing greater improvement than older boys (*p* = 0.057, Cohen’s *d* = 2.30). The post-hoc power analysis indicated a 99% chance of detecting a significant effect for all boys, 88% for younger boys and 56% for older boys, significant at the 5% level (two-tailed). There were no significant differences in balance for any participant.

**Table 6:** Movement Assessment Battery for Children standard scores.

Variable	All (*n =* 15)		Boys (*n =* 7)		Girls (*n =* 8)	
	Pre	Post	Pre	Post	Pre	Post
Total	9.33 (2.92)	10.00 (2.93)	7.86 (2.12)	8.86 (2.34)	10.63 (3.02)	11.00 (3.16)
Aiming and Catching	8.40 (2.13)	9.73 (2.40) *	8.71 (2.50)	9.86 (2.12)	8.13 (1.88)	9.63 (2.77)
Balance	10.80 (2.73)	9.93 (2.76)	9.29 (1.98)	9.43 (2.99)	12.13 (2.70)	10.38 (2.67)
Manual Dexterity	9.40 (3.94)	10.60 (3.60)	7.00 (2.45)	8.71 (2.63) **	11.50 (3.89)	12.25 (3.66)

Notes: Mean scores for the motor skills assessments at pre-test and post-test. Maximum score for each scale is 19. * = *p* < 0.06 ** = *p* < 0.01.

To further investigate the impact of AVGs on motor skills, regression analyses were conducted using playtime as a predictor. Analyses used total playtime over six weeks, as well as time spent playing each game. Overall, playtime significantly predicted total motor skills (R^2^ = 0.310, *p* = 0.031) for all participants. Follow up analyses found that total playtime was not significant for boys (R^2^ = 0.024), but was significant for girls (R^2^ = 0.506, *p* = 0.048). There was a trend towards a significant prediction of manual dexterity (R^2^ = 0.236, *p* = 0.067) for all participants. For individual games, the model to predict total motor skills was not significant; however, several games had trends towards significance. For all participants both Wii Play (*p* = 0.071) and Wii Sports Resort (*p* = 0.059) had a positive trend. This was also seen in girls, with positive trends found for Wii Play (*p* = 0.051) and Wii Sports Resort (*p* = 0.077), as well as for Just Dance 2 (*p* = 0.089). The model predicting manual dexterity for all participants (R2 = 0.636, *p* = 0.027) was significant. Both Wii Play (*p* = 0.005) and Wii Sports Resort (*p* = 0.051) significantly predicted manual dexterity scores. When analyzed singly, there were no significant results for boys. Although girls did not have a significant model predicting manual dexterity, Wii Play (*p* = 0.023) was still a significant predictor; additionally, Wii Sport Resort (*p* = 0.088) and Just Dance 2 (*p* = 0.072) had a trend towards a significant impact.

## 4. Discussion

Each of the four domains of physical literacy (physical activity behaviours, physical fitness, motor skills, and knowledge and understanding) were assessed in this study. Significant improvements however, were only found in two domains—motor skills, and knowledge and understanding. Improvements in motor skills were mixed; girl’s manual dexterity improved as their gameplay time increased, whereas boys significantly improved their manual dexterity regardless of gameplay. Additionally, manual dexterity (as opposed to balance or aiming and catching) was most closely related to overall motor skills. Knowledge and awareness in terms of motivation and enjoyment significantly improved for participants. Girls reported feeling less pressure, indicating the Wii had a positive impact on their attitude towards physical activity.

Physical activity behaviors, specifically average daily step count, did not change over the six-week intervention. Previous research with school based activity interventions have impacted school-time behaviours, but do not change activity behaviours outside of school [22]. This intervention took place at a childcare center—a structured environment outside of the home—and did not require children to change their daily routines. It is possible that their activity levels at the center may have been altered by the intervention, but similar to school-based interventions the effect did not carry over to their daily lives.

Physical fitness levels did not change over the duration of the study. Although participants reported lower levels of exertion at the post-test there was no change in their heart rate or distance walked in the 6MWT. Potentially participants gained a better understanding of how to rate their exertion throughout the study. Many adults have difficulty accurately rating their level of exertion [23], it is likely that children would experience similar difficulties. Conceivably, as participants engaged in more physical activity sessions their understanding of rating exertion improved, ultimately changing their responses. An alternative explanation for the lack of change in heart rate may be that the breaks in between playing games (loading times) allowed for children’s heart rates to lower before new games.

Boys and girls may take different approaches to the Wii games. Previous studies indicate boys are more likely to reduce unnecessary movements, thus being more efficient [13]. This may have impacted their manual dexterity, as smaller movements can sometimes be more effective for game success [13]. For example, in a basketball game, rather than completing a jumping and throwing motion, players can achieve the same results using a simple wrist flick. Regardless of game type or play duration, boys experienced improvements in manual dexterity, whereas girl’s improvements were related to the games that they played. It is probable that for all games boys were more apt use minimalist movements, thus all game play impacted their manual dexterity. Girls are less likely to reduce to minimalist movements for all games [13], and thus games that naturally use smaller movements (e.g., table tennis, cycling) as opposed to full body movements (e.g., dance based games) may have had a greater impact on their manual dexterity. However, these smaller movements do require less energy, which may also account for some of the decrease in exertion reported during the intervention [6]. 

Girls, as opposed to boys, are more likely to participate in games for fun or enjoyment and social interactions [24]. Rather than competition to win, girls generally engage in games for intrinsic reasons. The IMI subscale pressure is a negative predictor of intrinsic motivation, with greater scores indicating lower levels of motivation [18]. The present study found significant changes in the pressure scale for females, with a trend towards significance for all participants. When the goal is not to win the game, but rather to have fun, AVG gameplay reflects real-world game situations [13]. Unlike boys who use minimalist, efficient movements to increase performance, girls play more closely reflects standard gameplay movements.

### Limitations and Future Directions

Based on attendance at the Activity Centre some children had a greater opportunity for Wii play. Although all played a minimum of twice per week, the children who attended the Center more frequently received extra time when other children were unavailable to play in pairs [11]. Additionally, children were allowed to both self-select and change games during the play sessions. This flexibility was time consuming, resulting in children with less game playtime. The rationale for playing in pairs and allowing children to select their own games was to maximize ecological validity. Studies in a laboratory are often not “natural,” and participants may behave differently than in the real world [25]. By attempting to maximize ecological validity the responses of the participants typically mirrors real-world responses. As a trade-off the methodology is not as rigorous. Future research should aim to balance ecological validity and solid methodologies to accurately determine the effects of playing AVGs and their benefits in real world situations.

A limitation of many AVG systems, including the Nintendo Wii, is that motion is recorded through a handheld controller. While full body movements are encouraged they are not compulsory for game success. More experienced Wii players are able to utilize smaller wrist and arm movements while maintaining high levels of performance [13]. As children gained experience with the Wii they may have adopted this minimalist approach. Regardless, the data suggest that heart rate values and rates of perceived exertion remained consistent across the study, suggesting that our participants maintained similar movement levels.

## 5. Conclusions

Overall, playing Wii games produces some benefits to specific domains of physical literacy. Although authentic game play is still the best activity for children, AVGs are a good alternative for many individuals, providing a safe environment for children to develop basic physical literacy skills. Ultimately physical literate children are enabled to participate in a variety of activities throughout life. Though no change in daily activity or fitness levels after the Wii intervention were evident, there was an improvement in both motor skills and attitudes towards physical activity. Based on the differential impact of the games on various physical literacy skills, further investigation into the specific benefits of gameplay is warranted.
